# Economic value of AI-based MRI triage for Parkinson’s disease: a cost-benefit study in South Korea and the United States

**DOI:** 10.3389/fpubh.2025.1723829

**Published:** 2025-12-18

**Authors:** KyungYi Kim, Soohwa Song, Sung Sik Kim, Dong Hoon Shin, A-Leum Lee

**Affiliations:** 1 Department of Biohealth Industry, Graduate School of Transdisciplinary Health Sciences, Yonsei University, Seoul, Republic of Korea; 2 Heuron Co., Ltd., Seoul, Republic of Korea; 3 Severance Hospital, Seoul, Republic of Korea; 4 Department of Radiology, Soonchunhyang University Hospital Bucheon, Bucheon-si, Gyeonggi-do, Republic of Korea

**Keywords:** artificial intelligence, cost-benefit analysis, economic evaluation, magnetic resonance imaging, Parkinson’s disease, positron emission tomography

## Abstract

**Background:**

Early and accurate diagnosis of Parkinson’s disease (PD) remains a major clinical and economic challenge, particularly in settings where dopaminergic imaging, such as positron emission tomography (PET) scans, is limited by cost, availability, and patient access. Artificial intelligence (AI) has emerged as a promising tool to support magnetic resonance imaging (MRI)-based diagnosis of PD, but its economic value has yet to be fully evaluated.

**Methods:**

The AI model used in this study analyzes susceptibility map-weighted MRI to detect nigrosome-1 signal loss (the “swallow-tail sign”), providing objective support for early PD identification. We conducted a patient-level cost–benefit analysis (CBA) comparing current PET-based diagnostic pathways with an MRI-based AI triage strategy for PD. A total of 24 mutually exclusive diagnostic scenarios were modeled to capture variation in disease presence, AI accuracy, and PET access. The analysis was conducted from a societal perspective in South Korea and a healthcare system perspective in the United States, covering both short-term (1-year) and long-term (2025–2050) horizons. Sensitivity analyses and AI adoption rate scenarios (30, 65, 100%) were included.

**Results:**

In short-term analysis, AI-assisted diagnosis yielded net benefits of 9.3 million US dollars (USD) (South Korea) and 76.0 million USD (United States) under 30% adoption, which increased to 31.0 million USD and 253.2 million USD, respectively, under full AI adoption. Benefit–cost (B/C) ratios exceeded 1.4 in Korea and 1.3 in the U. S., and net benefit remained positive up to an AI unit cost of 226 USD in Korea and 1,506 USD in the U. S. The AI model also reduced PET use by over 31% through effective triage and enabled over 13,000 Korean PD patients to access PET who might otherwise have forgone it due to cost. Long-term projection (Korea only) indicated cumulative net savings of 2.5 billion USD by 2050 with gradually increasing AI adoption.

**Discussion:**

MRI-based AI triage for PD diagnosis is a cost-beneficial strategy with the potential to reduce unnecessary imaging and expand access among underserved populations. Particularly in health systems with limited PET availability, this approach may offer scalable economic and clinical advantages over time.

## Introduction

Idiopathic Parkinson’s disease (IPD) is a progressive neurodegenerative disease characterized by tremors, bradykinesia, rigidity, and postural instability (1). While the clinical differential diagnosis of parkinsonism is often straightforward, additional diagnostic work-up may be warranted in patients with atypical presentations or early/mild stages of disease. Current clinical guidelines suggest cranial magnetic resonance imaging (MRI) as an initial structural assessment and recommend functional imaging such as positron emission tomography (PET) with fluorodeoxyglucose (FDG-PET) or dopamine transporter single-photon emission computed tomography (DAT-SPECT) when clinically indicated, particularly for diagnostically uncertain or atypical symptoms (2). However, dopaminergic imaging modalities are limited by high costs, radiation exposure, long scan times, and restricted accessibility. In South Korea, despite one of the highest PET scanner densities worldwide, availability remains only 3.8 per million people (3).

Nigrosome-1, located in the dorsolateral substantia nigra pars compacta, is the earliest site of dopaminergic neuron degeneration in IPD. Loss of T2/susceptibility-weighted hyperintensity, also known as the “swallow-tail sign,” in nigrosome-1 has emerged as a sensitive and specific early imaging biomarker for IPD (4). Recent advances in high-resolution MRI sequences, particularly susceptibility map-weighted imaging (SMWI), have enabled reliable visualization of this region.

Building on this progress, artificial intelligence (AI)-based deep learning models have been developed to automatically detect and quantify nigrosome-1 abnormalities on SMWI MRI (5). Previous studies demonstrated that such models facilitate rapid and accurate quantification of nigral hyperintensity, support IPD diagnosis, and predict symptom severity, showing particularly high specificity (5, 6). When used as a triage tool, MRI-based AI quantification can potentially improve cost-effectiveness by restricting expensive, radiation-exposing FDG-PET to only those patients suspected of early IPD by the AI. Although no prior study has formally evaluated MRI-based AI as a PET triage mechanism, previous work has shown that deep-learning models substantially enhance MRI-based detection of nigrosome-1 abnormalities, providing a plausible rationale for using AI to guide downstream imaging decisions. Importantly, this triage role reflects AI’s function in enhancing MRI interpretation rather than replacing dopaminergic imaging. PET remains the confirmatory standard when diagnostic uncertainty persists in clinical practice. This approach may help streamline diagnostic workflows and reduce the burden on specialized imaging resources. However, the cost–benefit of such AI triage strategies may vary depending on the healthcare system, insurance structure, and diagnostic costs.

Despite its clinical promise, widespread adoption of AI-based diagnostic tools requires careful economic evaluation, particularly within publicly funded healthcare systems. Quantifying the trade-offs between diagnostic accuracy, healthcare costs, and resource allocation is essential to guide real-world implementation and reimbursement decisions.

Given the variability in healthcare systems, insurance structures, and diagnostic costs across countries, the cost–benefit of such AI triage strategies may differ significantly by setting. Therefore, this study aims to evaluate the economic value of implementing an MRI-based AI diagnostic strategy for Parkinson’s disease (PD) using cost–benefit analysis (CBA) in two national contexts: South Korea and the United States.

## Materials and methods

### Study overview

This study conducted a cost–benefit analysis (CBA) to evaluate the economic value of implementing an AI-assisted MRI triage strategy for the early diagnosis of IPD, compared to the conventional diagnostic strategy based on direct PET imaging. The analysis was performed from the societal perspective for South Korea and the healthcare system perspective for the United States. The model included a short-term (1-year) time horizon for both countries, and an additional long-term horizon (2025–2050) for South Korea to assess extended economic outcomes. The target population comprised adults aged 65 years or older who were clinically suspected of having Parkinson’s disease. Based on national demographics and previously reported incidence rates, the cohort was assumed to consist of 48,888 individuals in South Korea and 90,000 individuals in the United States. A patient-level simulation model was used to represent diagnostic and cost pathways for each individual within a hypothetical national cohort.

### AI model description

The AI-assisted diagnostic strategy in this study was based on Heuron IPD, a commercially available software developed by Heuron Co., Ltd. (Seoul, Republic of Korea), designed for automated nigrosome-1 assessment on SMWI (6–8). Neuroradiologists performed visual grading of the substantia nigra with reference to patients’ neurological examination findings, and discrepant cases were adjudicated through consensus review. The dataset was partitioned into training, validation, and internal test sets, and augmentation techniques were applied to improve robustness to variations in MRI acquisition. The model outputs quantitative indices including bilateral nigral hyperintensity maps, volumetric measures, and standardized Z-scores, providing objective support for early IPD assessment. Diagnostic accuracy parameters used in this study were sources from the software’s validated performance report. Similar deep-learning approaches have been used in other neurological contexts to detect subtle and pathognomonic visual or temporal features such as dynamic facial landmark patterns for depression or distributionally robust modeling of heterogeneous facial expressions, highlighting the broader technical relevance of feature-based AI diagnostics (9, 10).

### Intervention and comparators

Two strategies were compared: (1) the conventional functional-imaging pathway, in which patients for whom PET would normally be clinically considered undergo PET imaging for diagnostic confirmation, and (2) the AI-assisted MRI triage strategy, where MRI interpreted with AI is used to determine whether PET is necessary rather than assuming MRI is universally followed by PET. This modeling framework focuses on the subset of cases in which PET is clinically relevant due to diagnostic uncertainty, rather than implying universal PET use in all suspected PD patients. This approach allowed early-stage detection in high-confidence MRI-AI cases while reducing unnecessary PET procedures. The AI software was assumed to be applied at the point of MRI interpretation, prior to any PET decision.

### Model structure

The simulated cohort represents patients for whom PET may be clinically indicated based on guideline-consistent diagnostic uncertainty, and each individual in the simulated cohort was assigned to one of 24 mutually exclusive patient types, defined by combinations of five binary variables: (1) presence or absence of actual Parkinson’s disease, (2) presence or absence of economic burden to access PET imaging, (3) detection outcome of MRI (detected or not), (4) detection outcome of PET (detected, not detected, or not performed), and (5) whether AI was used in interpreting MRI. These 24 combinations represented all possible clinical and diagnostic scenarios under both strategies. Types 1–12 correspond to the AI-assisted strategy, while types 13–24 were assigned to the conventional PET-based pathway. The clinical characteristics of each type were identical between the two groups, but diagnostic workflows and associated cost structures varied. The analysis therefore focuses on diagnostic pathways among patients likely to undergo PET under standard care, rather than modeling PET use in all suspected PD patients. The AI adoption rate was modeled as a key variable determining the distribution between the two groups. For instance, with a 40% AI adoption rate, 40% of the population is distributed among types 1–12, and the remaining 60% is distributed among types 13–24. Each type’s cost and benefit trajectory were simulated based on their diagnostic path, accounting for early detection, misdiagnosis, delayed diagnosis, or avoided PET. Table 1 provides detailed definitions of all 24 patient types and diagnostic logic.

**Table 1:** Patient classification matrix by PD status, economic burden, diagnostic results, and AI usage.

Patient types	PD status	Economic burden	MRI detection	PET detection	AI usage	Notes
1	True	No	O	O	Yes	Additional AI cost
2	True	No	O	X	Yes	Additional AI cost
3	True	No	X	O	Yes	Additional AI cost; missed diagnosis causing delayed diagnosis costs
4	True	No	X	X	Yes	Additional AI cost; missed diagnosis causing delayed diagnosis costs
5	True	Yes	O	O	Yes	Additional AI cost; additional PET cost; early treatment cost saving
6	True	Yes	X	N/A	Yes	Additional AI cost
7	False	No	O	O	Yes	Additional AI cost; PET cost saving by triage
8	False	No	O	X	Yes	Additional AI cost; PET cost saving by triage
9	False	No	X	O	Yes	Additional AI cost
10	False	No	X	X	Yes	Additional AI cost
11	False	Yes	O	N/A	Yes	Additional AI cost
12	False	Yes	X	O	Yes	Additional AI cost
13	True	No	O	O	No	Same as comparator (conventional PET strategy)
14	True	No	O	X	No	Same as comparator (conventional PET strategy)
15	True	No	X	O	No	Same as comparator (conventional PET strategy)
16	True	No	X	X	No	Same as comparator (conventional PET strategy)
17	True	Yes	O	O	No	Same as comparator (conventional PET strategy)
18	True	Yes	X	N/A	No	Same as comparator (conventional PET strategy)
19	False	No	O	O	No	Same as comparator (conventional PET strategy)
20	False	No	O	X	No	Same as comparator (conventional PET strategy)
21	False	No	X	O	No	Same as comparator (conventional PET strategy)
22	False	No	X	X	No	Same as comparator (conventional PET strategy)
23	False	Yes	O	N/A	No	Same as comparator (conventional PET strategy)
24	False	Yes	X	O	No	Same as comparator (conventional PET strategy)

Types 1–12 reflect patients under the AI-assisted MRI triage strategy, while types 13–24 to the conventional PET strategy. “PD status” indicates whether the patient truly has PD, irrespective of diagnostic outcomes. This represents the ground truth status used for modeling accuracy and misdiagnosis. “Economic burden” refers to whether the patient faces significant financial strain when accessing diagnostic services; this affects PET utilization in the model. “MRI detection” represents whether PD is detected using MRI. “O” indicates a positive detection, “X” indicates non-detection. “PET detection” indicates whether PD is detected using PET imaging. “O” signifies detection, “X” signifies non-detection, and “N/A” indicates that PET was not performed. “AI use” is marked “Yes” if the patient followed the AI-assisted MRI triage strategy, and “No” if the conventional PET strategy was used. The cost consequence column describes the cost attribution for each case: whether AI-related costs were incurred, PET imaging was avoided, or if delayed diagnosis penalties applied. PD, Parkinson’s disease; PET, Positron Emission Tomography; MRI, Magnetic Resonance Imaging; AI, Artificial Intelligence. Shades are not related to its significance, rather blue means positive (sensitive or cost-saving) cases while red represents negative (additional costs or miss-diagnostics) situations.

### Input variables

Table 2 summarizes all key input parameters, including diagnostic test characteristics, initial probabilities of clinical conditions among the suspected PD population, direct medical costs for imaging or AI processing, annual treatment costs for early versus delayed management of PD, and relevant non-medical cost variables. AI sensitivity and specificity values were derived from a multicenter clinical trial across 10 tertiary care hospitals in South Korea (ClinicalTrials.gov Identifier: NCT4334902). Parameter values were derived from published literature, national statistics, and clinical trial data when available.

**Table 2:** Model input parameters used in the model.

	Variable types	South Korea	Source	USA	Source
1. Population characteristics					
V1.1	PD diagnostic rate among outpatients (%)	0.50	Assumed	0.50	Assumed
V1.2	PD population aged 65 + (N)	48,888	(16, 17)	90,000	(18)
V1.3	AI adoption rate (%)	0.30	Assumed	0.30	Assumed
V1.4	PET unaffordability rate (%)	0.30	Assumed	0.30	Assumed
2. Diagnostic accuracy					
V2.1	Sensitivity (AI)	0.943	(19)	0.943	(19)
V2.2	Specificity (AI)	0.917	(19)	0.917	(19)
V2.3	Sensitivity (PET)	0.932	(20)	0.932	(20)
V2.4	Specificity (PET)	0.857	(20)	0.857	(20)
3. Direct medical costs (USD)					
V3.1	PET scan cost (per scan)	735	(20)	2,587	(21)
V3.2	AI usage cost (per scan)	7	Assumed	100	Assumed
4. Indirect medical costs (USD)					
V4.1	Annual medical cost after early PD diagnosis	4,062	(22)	24,439	(23)
V4.2	Annual medical cost after delayed PD diagnosis	4,753	(22)	30,439	(24)
5. Non-medical costs (USD)					
V5.1	No. of persons per visit (including caregiver)	2	Assumed	N/A	
V5.2	Time for PET scan (hrs)	4	Assumed	N/A	
V5.3	Time for outpatient visit (hrs)	4	Assumed	N/A	
V5.4	Average hourly wage of aged 65+	14.6	(25)	N/A	
V5.5	Employment rate of aged 65 + (%)	0.396	(26)	N/A	
V5.6	Transportation cost (round trip)	18.2	(27)	N/A	

Sensitivity (AI) and specificity (AI) refer to the performance of the deep-learning algorithm applied to SMWI MRI image alone. These metrics do not represent combined MRI+PET performance. PET remains the confirmatory reference standard in current clinical practice. PD, Parkinson’s disease; PET, Positron Emission Tomography; MRI, Magnetic Resonance Imaging; AI, Artificial Intelligence; USD, United States Dollar; N/A, not applicable.

Specific variables included the sensitivity and specificity of MRI with and without AI assistance, PET detection performance, per-scan cost of PET, AI software licensing fee, and average outpatient consultation and transportation costs. In addition, estimates for employment rates and average hourly wages among individuals aged 65 years or older were used to calculate indirect costs such as time loss and caregiver burden.

Where appropriate, country-specific values were assigned separately for South Korea and the United States. In South Korea, both direct medical and non-medical costs (e.g., time and transportation) were included in the base-case analysis to reflect real-world burden. For the U.S. analysis, only direct medical costs were considered in 2025 U.S. dollars. The exchange rate applied for currency conversion weas 1,416.54 KRW per 1 USD, based on government forecasts for 2025 (11).

### Statistical analyses

We calculated the marginal economic impact of AI-assisted diagnosis compared to the conventional PET strategy by estimating per-patient marginal benefits (MB) and marginal costs (MC) across 24 patient types (Table 3). The analysis considered both direct medical costs (e.g., PET scans, AI processing, treatment) and indirect costs (e.g., productivity loss, transportation). All input variables referenced in the formulas (e.g., v1.1, v2.3) are detailed in Table 2. The net benefit (total benefit minus total cost) and the benefit–cost (B/C) ratio were computed by aggregating individual patient-level values weighted by their respective population proportions. In the Korean setting, we additionally simulated a long-term scenario model, projecting the cumulative net benefit through 2050, assuming AI adoption increases linearly to 80% within 10 years. This model incorporated both direct and indirect costs. By contrast, the U.S. analysis focused solely on direct medical costs, excluding non-medical elements due to limited data generalizability.

**Table 3:** Formulas and explanations of marginal benefits (MB) and marginal costs (MC) by patient types: comparing AI vs. PET diagnosis pathways.

Formulas for marginal benefits (MB) and marginal costs (MC)					Meaning	Patient types
MB1	Marginal benefit	Medical costs	Costs for PET	v1.2 * (1 − v1.4) * v1.3 * (1 − v2.1) * v3.1	Avoided PET costs due to false negatives identified by AI (without economic burden)	3, 4
MB2	Marginal benefit		Costs for PET	{v1.2 * (1 − v1.1)/v1.1} * (1 − v1.4) * v1.3 * v2.2 * v3.1	Avoided PET costs for true negatives correctly excluded by AI	7, 8
MC1	Marginal cost		Costs for PET	v1.2 * v1.4 * v1.3 * v2.1 * v3.1	Additional PET costs due to true positives identified by AI (with economic burden)	5
MC2	Marginal cost		Costs for PET	{v1.2 * (1 − v1.1)/v1.1} * v1.4 * v1.3 * (1 − v2.2) * v3.1	Additional PET costs due to false positives generated by AI	12
MC3	Marginal cost		AI processing costs	(v1.2/v1.1) * (1 − v1.4) * v1.3 * v3.2	AI usage cost for true positives	1, 2, 3, 4, 7, 8, 9, 10
MC4	Marginal cost		AI processing costs	v1.2 * v1.4 * v1.3 * v3.2	AI usage cost for false positives	5, 6
MC5	Marginal cost		AI processing costs	{v1.2 * (1 − v1.1)/v1.1} * v1.4 * v1.3 * v3.2	AI usage cost for false negatives	11, 12
MB3	Marginal benefit		Treatment costs	v1.2 * (1 − v1.4) * v1.3 * (1 − v2.1) * v2.3 * v4.1	Reduced treatment costs due to earlier diagnosis of true positives by AI	3
MC6	Marginal cost		Treatment costs	v1.2 * v1.4 * v1.3 * v2.1 * v2.3 * v4.1	Additional treatment cost for false negatives due to delayed diagnosis	5
MB4	Marginal benefit		Treatment costs	v1.2 * v1.4 * v1.3 * v2.1 * v2.3 * v4.2	Reduced treatment costs due to fewer false positives with unnecessary treatment	5
MC7	Marginal cost		Treatment costs	v1.2 * (1 − v1.4) * v1.3 * (1 − v2.1) * v2.3 * v4.2	Productivity loss due to delayed diagnosis in false negatives	3
MB5	Marginal benefit	Indirect costs	Time costs	v1.2 * (1 − v1.4) * v1.3 * (1 − v2.1) * v5.1 * v5.4 * v5.5 * v5.2	Avoided productivity loss for true positives diagnosed early	3, 4
MB6	Marginal benefit		Time costs	{v1.2 * (1 − v1.1)/v1.1} * (1 − v1.4) * v1.3 * v2.2 * v5.1 * v5.4 * v5.5 * v5.2	Avoided productivity loss for false positives correctly excluded	7, 8
MC8	Marginal cost		Time costs	v1.2 * v1.4 * v1.3 * (1 − v2.1) * v2.3 * v5.1 * v5.4 * v5.5 * v5.2	Productivity loss due to unnecessary care in false positives	5
MC9	Marginal cost		Time costs	{v1.2 * (1 − v1.1)/v1.1} * v1.4 * v1.3 * (1 − v2.1) * v2.3 * v5.1 * v5.4 * v5.5 * v5.2	Transportation cost for false negatives receiving PET confirmation	12
MB7	Marginal benefit		Transportation costs	{v1.2 * (1 − v1.1)/v1.1} * (1 − v1.4) * v1.3 * v2.2 * v5.1 * v5.6	Avoided transportation costs for true positives or false positives correctly excluded	7, 8
MC10	Marginal cost		Transportation costs	v1.2 * v1.4 * v1.3 * (1 − v2.1) * v2.3 * v5.1 * v5.6	Transportation cost for false positives undergoing unnecessary PET	5
MC11	Marginal cost		Transportation costs	{v1.2 * (1 − v1.1)/v1.1} * v1.4 * v1.3 * (1 − v2.1) * v2.3 * v5.1 * v5.6	Transportation cost for true positives receiving PET confirmation	12

Variables used in the formulas (e.g., v1.1, v.2.3) are defined in Table 2, which lists diagnostic parameters, cost components, and population probabilities. Each marginal benefit or marginal cost represents the unit-level difference between AI and PET strategies. PET, Positron Emission Tomography; AI, Artificial Intelligence; MB, Marginal Benefit; MC, Marginal Cost.

### Ethics approval and consent to participate

This study protocol was reviewed and approved by the Institutional Review Board (IRB) of Severance Hospital, Yonsei University (IRB number: 1-2025-0034). Written informed consent was waived by the IRB due to the retrospective and de-identified nature of the data.

## Results

### Short-term economic outcomes

Table 4 summarizes the short-term economic impact of adopting AI-assisted diagnosis for PD across three levels of adoption (30, 65, 100%) in both South Korea and the United States. In Korea, where a societal perspective was taken, the net benefit was estimated at 9.29 million US dollars (USD) under 30% AI adoption and increased to 30.97 million USD at full adoption. The benefit–cost (B/C) ratio was 1.48, indicating consistent efficiency gains. In the United States, where only direct medical costs were considered, the estimated net benefit rose from 75.96 million USD at 30% AI adoption to 253.19 million USD at 100% adoption, with B/C ratios of 1.36. Notably, the break-even point for AI unit cost, where net benefit becomes zero, was calculated to be approximately 226 USD in Korea and 1,506 USD in the United States. These thresholds suggest that the AI model remains cost-effective even with considerable implementation costs. Because the PET unaffordability rate (base case: 30%) is a key structural assumption in the model, sensitivity analysis was performed across a wide range (0–100%). Net benefit remained positive throughout, varying from 10.90 million USD at 0% unaffordability to 5.53 million USD at 100%, corresponding to 117.3 to 59.5% of the base-case estimate. These results collectively suggest that AI integration into PD diagnostic pathways delivers substantial short-term economic benefits in both national settings, with increasing returns as adoption scales.

**Table 4:** Short-term cost–benefit results of AI adoption for PD diagnosis in Korea and the U.S.

(Unit: USD)	South Korea			United States		
	AI (30%)	AI (65%)	AI (100%)	AI (30%)	AI (65%)	AI (100%)
MB1	430,206	932,113	1,434,020	2,786,975	6,038,446	9,289,917
MB2	6,918,769	14,990,666	23,062,562	44,821,405	97,113,044	149,404,683
MC1	184,374	399,477	614,580	1,194,418	2,587,905	3,981,393
MC2	269,445	583,797	898,149	1,745,527	3,781,974	5,818,422
MC3	144,953	314,064	483,176	3,780,000	8,190,000	12,600,000
MC4	31,061	67,299	103,538	810,000	1,755,000	2,700,000
MC5	31,061	67,299	103,538	810,000	1,755,000	2,700,000
MB3	2,215,379	4,799,989	7,384,598	24,537,822	53,165,280	81,792,738
MC6	15,707,540	34,033,003	52,358,465	173,978,690	376,953,827	579,928,965
MB4	18,381,227	39,825,992	61,270,757	216,692,063	469,499,470	722,306,877
MC7	2,592,474	5,617,028	8,641,581	30,562,083	66,217,847	101,873,610
MB5	27,050	58,608	90,166			
MB6	435,030	942,564	1,450,099			
MC8	178,748	387,288	595,828			
MC9	178,748	387,288	595,828			
MB7	342,145	741,314	1,140,483			
MC10	140,583	304,597	468,611			
MC11	140,583	304,597	468,611			
Net benefit	9,290,818	20,130,105	30,969,392	75,957,548	164,574,686	253,191,825
B/C ratio	1.48			1.36		

### Projected reduction in PET scans

**Figure 1:**
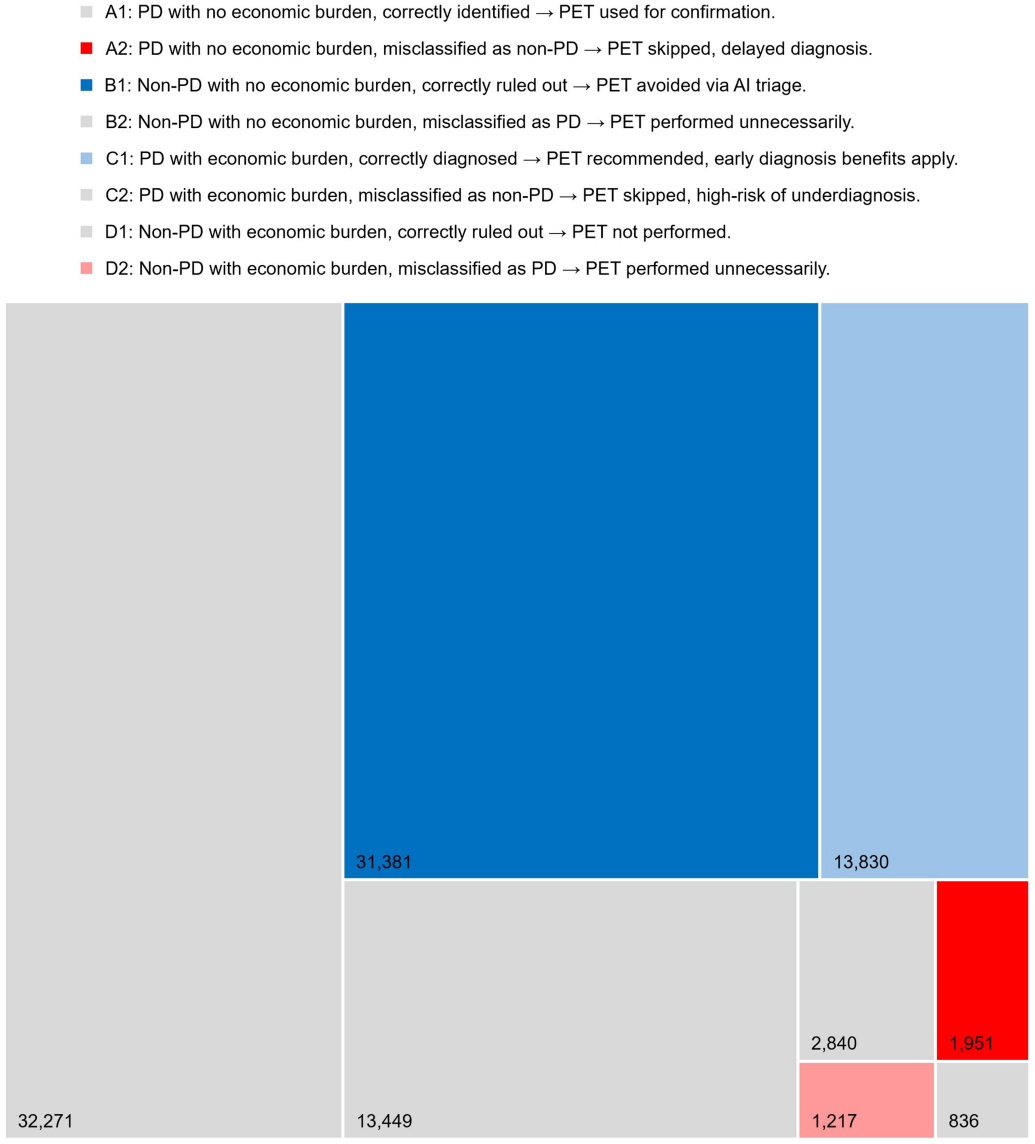
PET scan impact by patient subgroup (A1-D2) defined by PD status, economic burden, and sensitivity.

Figure 1 illustrates the distribution of patients across diagnostic subgroups under the AI-assisted pathway, with implications for PET scan utilization. Among a total of 97,776 patients assessed, 31,381 (32.1%) in group B1 represent avoided PET scans due to AI effectively ruling out non-PD cases, highlighting the model’s triage capability. 13,830 (14.1%) in group C1 correspond to PD patients who were correctly identified by AI but may have previously forgone PET imaging due to economic burden; under the AI-guided strategy, these individuals receive recommended PET scans reimbursable, enabling early diagnosis and timely intervention. In contrast, 1,951 (2.0%) in group A2 represent missed PD cases where PET scans were skipped due to AI misclassification, posing a risk of delayed diagnosis. Additionally, 1,217 (1.2%) in group D2 reflect PET scans unnecessarily conducted on non-PD patients misclassified as PD, contributing to potential overuse and excess cost. In the Korean clinical setting, PET is routinely recommended as the confirmatory step when diagnostic uncertainty persists, and MRI alone rarely determines final diagnosis. Therefore, the modeled cohort reflects patients for whom PET would normally be offered within this PET-eligible group, economic barriers represent the primary real-world reason for forgoing PET.

### Long-term projections through 2050 (Korea only)

**Figure 2:**
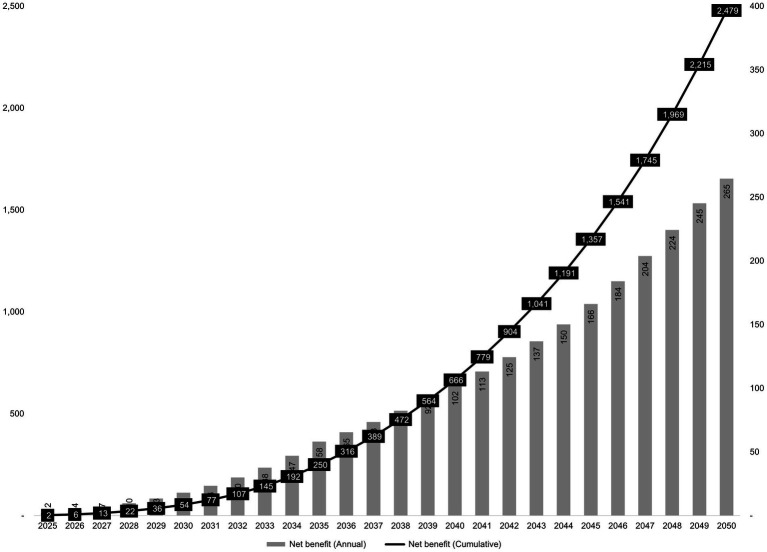
Projected annual and cumulative net benefits of AI-assisted diagnosis in Korea, 2025–2050 (unit: million USD).

Based on Korea’s projected PD prevalence and population aging trends, we estimated the annual and cumulative net benefit of implementing the AI-based diagnostic support tool over a 26-year horizon (2025–2050). The results indicated that the annual net benefit starts at 2.1 million USD in 2025 and increases steadily, reaching 264.7 million USD in 2050. Cumulatively, the net benefit is projected to amount to 2.5 billion USD over the 26-year period (Figure 2).

## Discussion

AI has emerged as a key enabler of diagnostic innovation, particularly in imaging-heavy diseases like PD, where early detection is crucial yet often delayed due to accessibility or cost constraints. Our study aimed to quantify the economic value of implementing an MRI-based AI triage system for PD diagnosis. Using CBA, we modeled patient-level outcomes across 24 diagnostic scenarios and assessed both short-and long-term impacts from national perspectives in South Korea and the United States.

Importantly, this modeling framework reflects the real-world clinical context in which PET is not universally performed but is typically reserved for diagnostically uncertain cases. To ensure the model aligned with actual practice, we referenced national Korean data indicating that approximately 20,000 PET scans are performed annually for Parkinsonism-related diagnostic evaluation. Thus, the model focused on the clinically relevant subgroup for whom PET would reasonably be considered, rather than assuming universal PET use across all suspected PD patients.

Furthermore, if future neuroprotective or disease-modifying therapies become available, early diagnostic confirmation will become substantially more valuable. In such a scenario, the AI-assisted MRI triage strategy evaluated in this study could function as a cost-effective early screening tool, particularly for early-stage PD. This highlights that the economic utility of AI could be even greater in future treatment landscapes than estimated in this analysis.

We developed a granular modeling framework that categorized into 24 mutually exclusive patient types, reflecting variations in disease presence, diagnostic pathways, economic burden, and AI detection outcomes. This structure enabled nuanced simulation of AI’s dual effects of reducing unnecessary imaging while expanding early access among patients who would have otherwise forgone PET due to cost barriers. The AI strategy was evaluated under both short-term (1-year) and long-term (2025–2050) horizons. For South Korea, we adopted a societal perspective, incorporating both medical and non-medical costs (e.g., transportation, productivity loss), while the U.S. model used a healthcare system perspective that included direct medical costs only. Adoption rates of 30, 65, and 100% were modeled to reflect gradual technology diffusion, and key assumptions were stress-tested through sensitivity analysis. In particular, varying the PET unaffordability rate from 0 to 100% did not change the direction of the results, and net benefit remained positive across all scenarios, underscoring the robustness of the model’s economic conclusions.

Our findings suggest that the implementation of an MRI-based diagnostic system can yield substantial economic benefits. In the Korean context, where societal costs are considered, the AI-assisted strategy resulted in a net savings of 9.3 million USD under 30% adoption and 31.0 million USD at full uptake. The B/C ratio remained above 1.4, supporting the strategy’s cost-efficiency. Importantly, the AI model triaged over 31% of patients out of PET imaging and also enabled PET access for over 13,000 PD patients who previously may have been excluded due to financial limitations. The long-term simulation (2025–2050) revealed even greater economic value, with cumulative net savings projected to reach 2.5 billion USD by 2050 under gradually increasing AI adoption in Korea. This suggests that the cost-effectiveness of AI not only persists but amplifies over time, particularly in aging societies with rising PD prevalence. These findings position AI as a scalable solution to address both access and cost challenges in neurodegenerative disease diagnostics. Previous studies have shown that AI aids cost-saving and improves healthcare outcomes in acute stroke and multiple sclerosis patients (12–14). Although a prior study has evaluated the cost-effectiveness of early PD detection using non-wearable sensors (15), to our knowledge this is the first economic evaluation of AI-based diagnostic software specifically for PD. This reinforces the novelty and policy relevance of our findings.

Despite the promising economic results, several limitations must be acknowledged. First, the model relied on assumptions for key parameters such as the proportion of patients who forgo PET imaging due to economic barriers and the long-term medical costs associated with delayed PD diagnosis. While these inputs were informed by existing literature, real-world validation is essential to refine these assumptions. In addition, the model focused on patients for whom PET would normally be recommended in the Korean clinical setting. Individuals who do not undergo PET because the diagnosis appears clinically obvious were not included, as such cases are uncommon in real-world practice. Second, AI performance metrics (e.g., sensitivity and specificity) were drawn from controlled trial settings. In clinical reality, diagnostic accuracy may vary depending on MRI protocol heterogeneity, institutional infrastructure, and radiologist expertise. Future prospective studies are needed to evaluate how AI tools perform when deployed across diverse healthcare environments. Third, the model adopted a simplified linear trajectory for AI uptake and assumed stable reimbursement and pricing structures over 25 years. Although real-world technology diffusion often follows nonlinear patterns such as S-curve adoption, empirical data on long-term uptake of AI-assisted PD imaging are currently unavailable. Therefore, a linear trajectory was applied as a conservative and transparent baseline assumptions. Additionally, while the U.S. model included only direct medical costs, broader societal costs such as caregiver burden and transportation could further improve the cost-effectiveness profile if included. Future research should also examine the integration of AI tools within clinical decision support systems and assess patient-centered outcomes, such as time to diagnosis, satisfaction, and long-term quality of life. Incorporating stakeholder perspectives, including physicians, payers, and patient advocacy groups, will be critical to support real-world adoption and scale-up.

In conclusion, this study demonstrates that MRI-based AI triage for PD is not only clinically promising but also economically viable. By reducing unnecessary PET scans and enabling access for underserved patients, AI can help optimize diagnostic pathways in both the short and long term. South Korea, with its rapidly aging population and robust imaging infrastructure, represents an ideal environment for early adoption. With further validation and policy alignment, this approach may serve as a replicable model for other nations aiming to modernize neurodegenerative disease diagnostics.

## Data Availability

The original contributions presented in the study are included in the article/supplementary material, further inquiries can be directed to the corresponding author.
